# Correction

**DOI:** 10.1080/13880209.2025.2580182

**Published:** 2025-10-25

**Article title:** Traditional Chinese medicine for breast cancer treatment: a bibliometric and visualization analysis

**Authors:** Jun Yuan, Yun Liu, Tiantian Zhang, Cheng Zheng, Xiao Ding, Chuanrong Zhu, Jing Shi and Yi Jing

**Journal:** *Pharmaceutical Biology*

**Bibliometrics:** Volume 62, Number 1, pages 499–512

**DOI:** https://doi.org/10.1080/13880209.2024.2359105

This paper contained errors mentioned below when the above-mentioned article was first published online.

1. The name “Taiwan” present in the sentences starting with “In English publications, China…” and “Among the top 10 countries/regions…” in the second paragraph of sub-section “National publications” was incorrect. The name “Taiwan” has been updated as “Taiwan region of China” in both sentences.
2. There was an error in Figure 3. Figure 3 has been updated now.

The article has been republished online after rectifying these errors and the updated Figure 3 has been displayed below.

**Figure 3.**
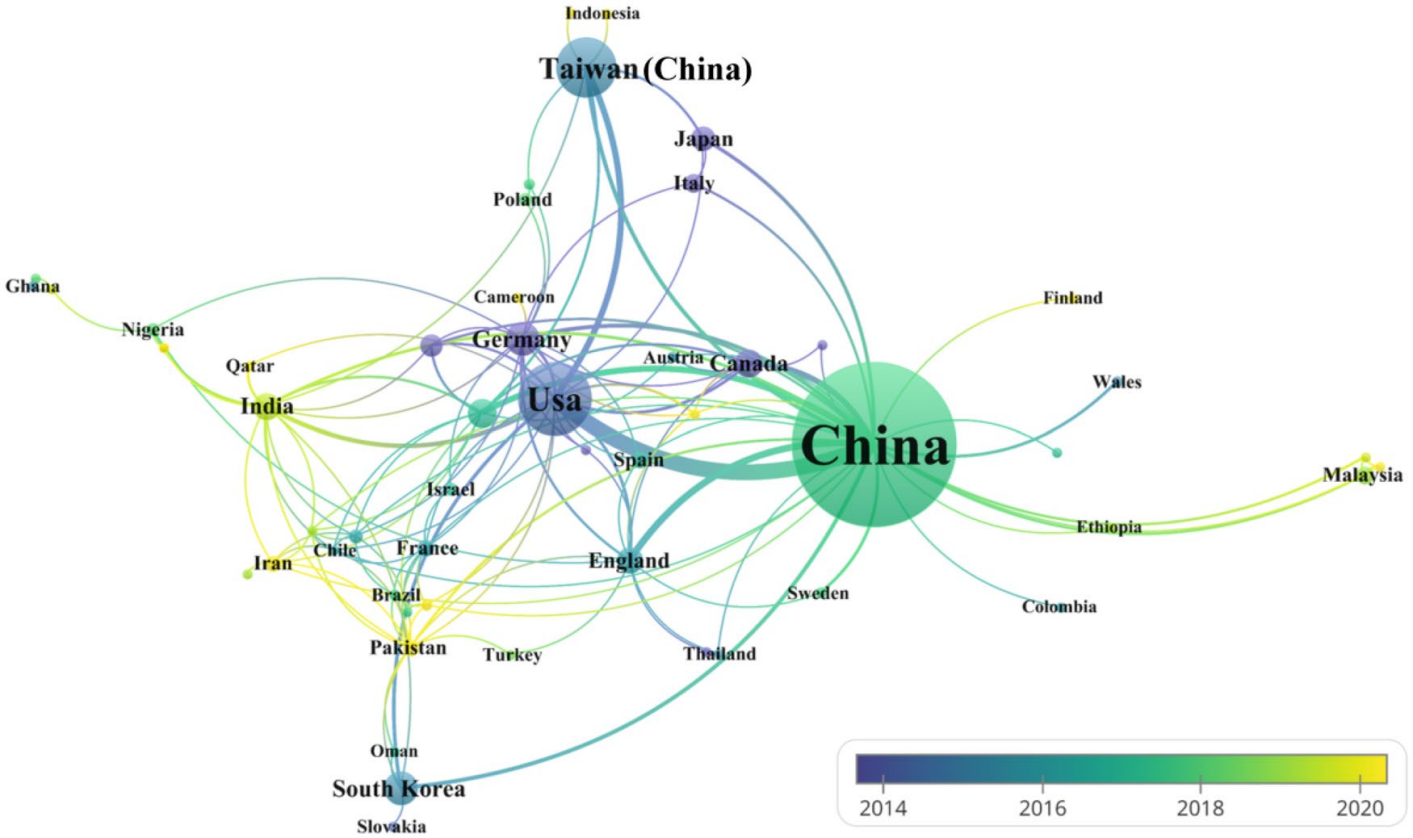
National collaboration network diagram.

